# Germ Cell Tumors: Updates on Epidemiology, Biology, and Treatment Considerations

**DOI:** 10.1155/2018/4821084

**Published:** 2018-08-19

**Authors:** Aditya Bagrodia, Costantine Albany, Timothy A. Masterson

**Affiliations:** ^1^Department of Urology, UT Southwestern Medical Center, Dallas, TX, USA; ^2^Division of Medical Oncology, Indiana University Medical Center, Indianapolis, IN, USA; ^3^Department of Urology, Indiana University Medical Center, Indianapolis, IN, USA

Germ cell tumors (GCTs) are the most common solid tumors in young men. GCTs represent a rare oncologic success story with 5-year cure rates exceeding 98% for early-stage tumors and 80% for patients with metastatic disease, attributable to the integration of surgery, cisplatin-based chemotherapy, and radiation therapy. Despite the excellent clinical outcomes in testicular GCTs, significant challenges and opportunities exist, as GCTs represent the most life years lost for nonpediatric malignancies [1].

There is certainly room for improvement in managing patients with germ cell tumors, and it is critical that the urology, oncology, and radio-oncology communities do not rest on the laurels of past successes. We must understand and modify socioeconomic differences that underpin germ cell tumor epidemiology and outcomes in order to narrow treatment gaps.

Special considerations for patients with pediatric and adolescent GCTs must also be recognized to improve outcomes among this subgroup. In this special issue, Amatruda et al. review the unique considerations of pediatric patients with GCTs, including demographic factors, histologic characteristics, treatment specifics, and emerging molecular data. Saltzman and Cost provide insights in treating adolescents with GCTs, including clinicopathologic outcomes, psychosocial support requirements, fertility and hypogonadism concerns, and transitional care needs.

Further, in this issue, considerable attention is appropriately dedicated to mitigating treatment-associated morbidity in patients with GCTs. Minimizing short, intermediate, and long-term treatment-related toxicity is mandatory for these young cancer survivors who have a whole life to live. Fung and colleagues comprehensively characterize complications associated with GCT treatment. The incidence and pathophysiology underlying common adverse effects such as cardiovascular disease, secondary malignancies, Raynaud's phenomenon, and neurotoxicity are reviewed, as are recent developments in understanding genetic predisposition for adverse consequences and ways to ameliorate them. Huddart and Reid describe the clinical experience with adjuvant chemotherapy for patients with high-risk Stage I nonseminomatous germ cell tumors. Supporting evidence for why one cycle of bleomycin, etoposide, and cisplatin (BEP) in the adjuvant setting is emerging as the optimal adjuvant chemotherapy regimen for patients with stage IB disease is provided in the context of preserving oncologic control and minimizing dose-dependent chemotherapy-associated adverse effects.

On the surgical end of the spectrum, Daneshmand and colleagues provide the rationale and early experience with retroperitoneal lymph node dissection (RPLND) for patients with small volume metastatic seminoma in an effort to avoid toxicities associated with either radiotherapy or chemotherapy, which are the treatment modalities typically used to manage these patients. In another effort dedicated to minimizing treatment-related morbidity, Pierarazio et al. describe the state of the literature concerning techniques and outcomes for robotic-assisted retroperitoneal lymph node dissection. Importantly, the authors note importance of ensuring oncologic outcomes is not compromised as this technology continues to develop. Cary and Masterson delineate the role of modified template retroperitoneal lymph node dissection in the primary and postchemotherapy setting, again demonstrating the commitment to avoid treatment toxicity, including retrograde ejaculation following RPLND while maintaining excellent cancer control. The authors review various templates and essential patient-selection characteristics, particularly when considering modified templates in the postchemotherapy setting.

Among patients with cisplatin-resistant disease, Feldman and colleagues discuss the ongoing clinical trial of conventional-dose salvage chemotherapy versus high-dose chemotherapy with stem cell support (NCT02375204) and the background literature supporting the need for this important trial. Chovanec and colleagues report on the preclinical work suggesting a role for immunotherapy in GCTs as well as early clinical experience in this exciting arena.

Last but certainly not least, Hamilton et al. describe their experience with risk-adapted surveillance in patients with GCT. This exciting research aims at individualizing the intensity of follow-up for patients with early-stage GCT to reduce both radiation exposure and costs associated with long-term surveillance.

In conclusion, it is our privilege to guest edit this special issue on germ cell tumors that highlights contemporary work in the field of GCTs. We are grateful to the experts in the field that provided their invaluable insight. This particular issue makes us optimistic that the future is bright for the optimal and refined management of patients with germ cell tumors.

*Aditya Bagrodia* *Costantine Albany* *Timothy A. Masterson*
